# The association between muscular strength and depression in Korean adults: a cross-sectional analysis of the sixth Korea National Health and Nutrition Examination Survey (KNHANES VI) 2014

**DOI:** 10.1186/s12889-018-6030-4

**Published:** 2018-09-15

**Authors:** Mee-Ri Lee, Sung Min Jung, Hyuk Bang, Hwa Sung Kim, Yong Bae Kim

**Affiliations:** 1 Department of Preventive Medicine & Institute of Occupational and Environmental Medicine, Soonchunhyang University College of Medicine, 30, Suncheonhyang 6-gil, Dongnam-gu, Cheonan-si, Chungcheongnam-do Republic of Korea; 2 Department of Surgery, Inje Univ. Ilsan Paik Hospital, 170 Juhwa-ro, IlsanSeo-gu, Goyang-si, Gyeonggi-do 10380 Republic of Korea

**Keywords:** Hand strength, Depression, Quality of life

## Abstract

**Background:**

There are conflicting researches on the relationship between muscular strength and depression, the most common mental illness. There is no study of relationship between muscular strength and depression using national data from young adults to seniors. For example, there has not been a study done explaining mediating pathways among the influences of handgrip strength on depression. Here, we conducted survey for the association between relative handgrip strength and depression and explain mediated pathways for quality of life.

**Methods:**

A cross-sectional study was administered to 4298 Korean adult subjects, aged 19–80 years, based on the 6th Korean National Health and Nutrition Examination Survey (KNHANES VI) of 2014. Handgrip strength reported as the average with each hand. The relative handgrip strength is defined as the handgrip strength divided by the body mass index (BMI). We performed analysis for all subjects and age groups (young adult, middle-aged, and elderly). We analyzed the association using multivariate linear regression and logistic regression. We also conducted mediation analysis for quality of life, which was measured by the EuroQol Five-Dimension Questionnaire (EQ5D).

**Results:**

After adjusting for covariates, handgrip strength was inversely associated with the PHQ-9 score (*P* < 0.05). The odds ratios (OR) of depression symptoms were statistically significant for participants in the first and second quartile of handgrip strength compared to those with the highest quartile in entire sample, young adult, middle-aged, and elderly. There was about a 50% mediation effect of EQ5D in the relationship between handgrip strength and depression.

**Conclusions:**

Using a large national sample, our results found that lower handgrip strength is associated with an increased risk of depression in Korean adult (young adult, middle-aged, and elderly). Our results suggest that increasing muscular strength may prevent depression in Korean adults.

**Electronic supplementary material:**

The online version of this article (10.1186/s12889-018-6030-4) contains supplementary material, which is available to authorized users.

## Background

Depression is a common mental disorder, and about 350 million people suffer from depression all over the world [1]. Depression was selected by the World Health Organization as the largest contributor to global disability (7.5% of all disabled people in 2015) [2]. Depression is also the leading cause of suicide deaths, which happens at a rate of 800,000 annually [2]. Depression is an important issue in Korea because, according to the Organization for Economic Cooperation and Development (OECD) Health Statistics 2017, Korea’s suicide rate was the highest, and an average of 28.7 people per 100,000 committed suicide in 2013, about 2.4 times the OECD average of 12.1 [3].

In a recent epidemiologic study, handgrip strength was a very simple and noninvasive measure of upper limb strength and an important marker of physical health [4]. As a physical fitness test, handgrip strength is an excellent predictor of short- and long-term mortality, more than muscle mass, and an indicator of nutritional status among hospitalized patients [5].

For older adults, epidemiologic studies have showed that low muscle strength is associated with mental health such as worse cognitive state and higher depressive symptoms [6, 7]. Longitudinal studies have shown that a decreasing handgrip strength is related with depression for subjects aged 40–79 years in Japan [8] and older adults in England [9]. Ortega et al. [10] found that higher muscle strength in adolescents was associated with a 20–30% lower risk of death from suicide for Swedish male in prospective cohort study. However, some studies of participants aged 70 years or older did not find a significant association with depression [11]. Another study also found that changes in handgrip strength did not predict changes in depression [12]. Thus, the association of muscle strength with depression remains controversial. Although most previous studies focused on the elderly or late midlife, little has been investigated about muscle strength on the depression of general adults.

Therefore, we conducted survey for the association between handgrip strength and depressive symptoms using the 6th Korean National Health and Nutrition Examination Survey (KNHANES VI). In addition, we analyzed the mediation effect of quality of life between handgrip strength and depression, because some studies have suggested that muscular strength, mediated by quality of life, influences depression [8].

## Methods

### Participants

KNHANES has been implemented at the national level from the Korea Center for Disease Control (KCDC) since 1998 and conducts direct physical examinations, clinical and laboratory tests, personal interviews and related measurements [13, 14]. The survey has been changed every year since 2007 [13, 14]. Population and Housing Census 2010 and new apartment data were used to extract samples [13, 14]. Since the foreign households were excluded from the survey, ethnicity was only for Koreans [13, 14]. Of the approximately 200,000 primary sampling units (PSUs), 192 PSUs were selected and 20 families were selected as the final target through systematic sampling of each PSU consisting of 60 households on average [13, 14]. Each KNHANES wave contains about 10,000 individuals of new samples [13, 14]. The final step of the selection is in the home selected for all members aged 1 and over to participate [13, 14]. The KNHANES’s overall response rate is targeted at 75% [13, 14]. Further details of KNHANES can be found elsewhere [13, 14].

**Fig. 1:**
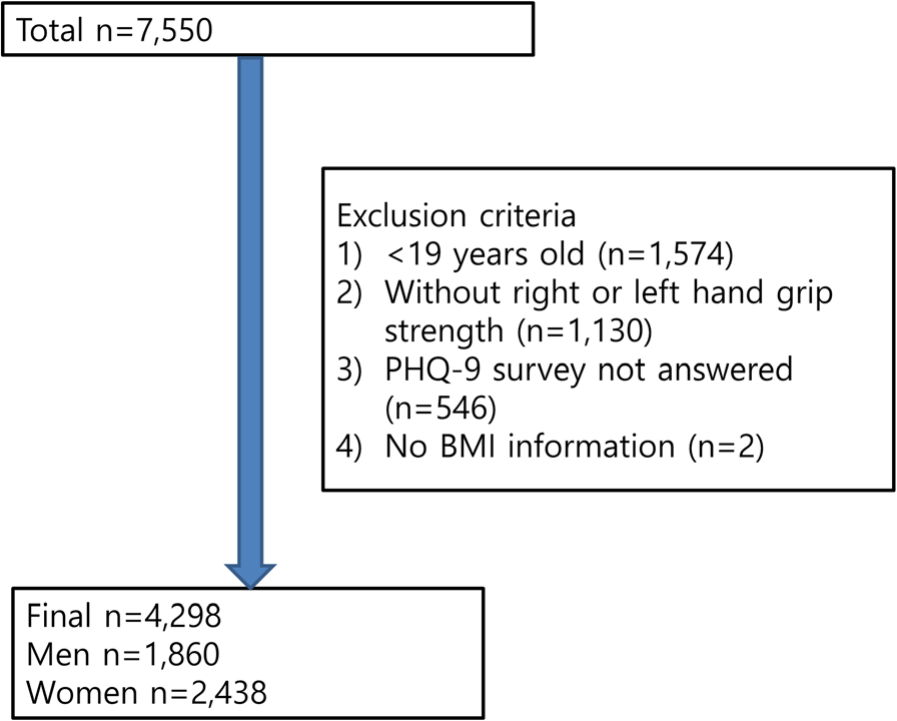
Participant flow diagram illustrates the number of those excluded and the number of data points analyzed

We used data in KNHAES VI (2014). Exclusion criteria were those who were under the age of 19 years (*n* = 1574), those data without handgrip strength for the right or left hand (*n* = 1130), those who did not have data for depressive symptoms (*n* = 546), and those who did not have data for body mass index (BMI) (*n* = 2). The final sample number was 4298 (Fig. 1).

The age range of the subjects was 19–80 years and the subjects were divided into three groups (young adult (19-39 years), middle aged (40–59 years), and elderly (60–80 years)).

### Assessment of covariates

A health questionnaire was used to obtain information on age (years), sex (men, women) and educational status (≤ elementary school, middle school, high school, ≥ university). Subjects were divided into quartiles based on the household income: less than 680,600 won (KRW), 680,600-1,480,399 won (KRW), 1,480,400–2,499,999 won (KRW), and greater than 2,500,000 won (KRW). A skilled health technician calculated body mass index (BMI) by measuring body weight (kg) and height (cm) according to standardized procedures for all participants. (BMI = weight/ (height) ^2^).We divided participants into underweight (BMI < 18.5), normal weight (18.5 ≤ BMI < 25), overweight (25 ≤ BMI < 30), obese (30 ≤ BMI) group [15]. The weight status of the subjects according to sex, age group, and presence or absence of depression was in Additional file 1: Table S1. The participants were divided into three groups (never smokers, past smokers, or current smokers). Alcohol drinkers were defined as “yes” for participants who had consumed at least one glass of alcohol every month over the last year. The physical activity among participants was defined as those who had performed exercise for least 2 h and 30 min per week, at a medium intensity of physical activity, high intensity physical activity for 1 h and 15 min, or a combination of medium and high-intensity physical activity (1 min of high-intensity = 2 min of medium intensity).

### Handgrip strength (HGS)

In this study, HGS was measured by a handgrip strength dynamometer (TKK 5401; Takei Scientific Instruments Co., Ltd., Tokyo, Japan) and detailed measured of HGS could be found elsewhere [16]. Measures of handgrip strength were reported as the average of the three measurements with either hand. The relative handgrip strength is defined as the handgrip strength divided by BMI.

### Patient Health Questionnaire-9 (PHQ-9)

KNHANES VI collected the PHQ-9 to estimate the degree of depression in participants. The PHQ-9 was based on self-reported measures and is useful for screening major depressive disorder [17]. The tool scores each of the nine items matching the Diagnostic and Statistical Manual of Mental Disorders, Fourth Edition criteria as “0” to “3” [17]. PHQ-9 has been translated into Korean and was available for free download from the PHQ website (https://www.phqscreeners.com/) [18]. The threshold for selecting depressive disorders using the Korean version of PHQ-9 was 5 [19].

### EuroQol Five-Dimension Questionnaire (EQ5D)

EQ5D was a tool for measuring quality of life and consists of five descriptive sections [20, 21]. More information could be found elsewhere [16].

### Statistical analyses

Continuous and categorical variables were expressed as the mean ± standard deviation and n (%), respectively. Differences in general characteristics according to depression were compared using the Student’s t-test or Chi square test, as appropriate in entire sample and age group (young adult (19-39 years), middle aged (40–59 years), and elderly (60–80 years)).

We analyzed the association between relative handgrip strength and depression (PHQ-9) using multivariate linear regression. We conducted model 1 (adjusted age and sex) and model 2 (adjusted age, sex, household income, alcohol consumption, smoking, body mass index, and physical activity). Logistic regression analysis was performed to examine associations between quartile of relative handgrip strength with presence of depression in entire sample and age group (young adult (19-39 years), middle aged (40–59 years), and elderly (60–80 years)).

We performed complex sample analyses using weight and further details of these weights can be found in the KNHANES VI (https://knhanes.cdc.go.kr/knhanes/main.do) [13, 21]. We used the “medeff” module in Stata [22] to assess whether EQ5D mediates the association between handgrip strength and presence of depression.

We performed sensitivity analyses dividing the total sample by sex.

We performed statistical analysis using STATA version 13 (Stata Corp., College Station, TX, USA) and found a significance level of 0.05 or less. Graph was performed using the R program version 3.3.3 (The Comprehensive R Archive Network: https://cran.r-project.org/) for Windows.

## Results

Among 4252 participants, 1835 (43.16%) were men. The average age was 44 years (range, 19–80).

The prevalence of depression in this study was 20.7% (men 15.7%, women 25.7%).

Table 1 showed that general characteristics for entire sample and age group. Age, sex, education, household income, diagnosis of depression for entire sample and all of age groups; smoking for entire sample, young adults, and elderly; alcohol consumption for elderly; BMI for entire sample, young adults, and middle-aged differed significantly between the depression and non-depression group.

**Table 1:** General characteristics of study population for age group and depressive symptoms (*n* = 4298)

	Total			Young adult (*n* = 1320)			Middle aged(*n* = 1561)			Elderly(*n* = 1417)		
	PHQ-9 < 5	PHQ-9 ≥ 5	*P*	PHQ-9 < 5	PHQ-9 ≥ 5	*P*	PHQ-9 < 5	PHQ-9 ≥ 5	*P*	PHQ-9 < 5	PHQ-9 ≥ 5	*P*
Sex, n(%)												
Men	1585(53.7)	278(38.2)	< 0.001	444(55.4)	108(40.3)	< 0.001	578(53.4)	80(37.9)	< 0.001	560(50.9)	90(33.3)	< 0.001
Women	1828(46.3)	610(61.9)		538(44.6)	230(59.7)		725(46.6)	178(62.1)		565(49.1)	202(66.7)	
Education (years), n(%)												
≤ 6	698(13.5)	251(19.2)	< 0.001	6(0.5)	4(1.5)	0.104	116(8.0)	42(14.5)	0.015	576(50.6)	205(69.4)	< 0.001
6–9	382(9.5)	77(7.4)		19(1.8)	9(3.1)		191(13.8)	36(11.7)		172(15.2)	32(11.4)	
9–12	1161(39.0)	294(40.5)		397(45.1)	153(49.9)		535(41.8)	103(42.9)		229(21.1)	38(13.8)	
12<	1167(38.0)	265(33.0)		559(52.6)	172(45.6)		460(36.4)	77(31.0)		148(13.1)	16(5.4)	
Non-response	2	1		1	0		1	0		0	1	
Income, n(%)												
1Q	518(11.4)	242(21.2)	< 0.001	56(6.2)	35(11.9)	0.028	79(5.7)	39(14.1)	< 0.001	383(33.7)	168(54.5)	< 0.001
2Q	845(23.8)	220(27.3)		219(23.9)	77(24.4)		288(21.3)	79(33.2)		338(29.3)	64(25.7)	
3Q	1028(32.1)	213(26.3)		374(35.7)	112(32.1)		444(34.8)	68(27.0)		210(19.3)	33(11.5)	
4Q	1013(32.7)	206(25.1)		333(34.2)	112(31.6)		491(38.3)	69(25.7)		189(17.7)	25(8.3)	
Non-response	6	7		0	2		1	3		5	2	
Smoking, n(%)												
Non-smoker	2033(56.8)	553(59.2)	< 0.001	615(60.0)	203(58.1)	0.008	780(53.8)	158(57.5)	0.245	638(57.1)	192(64.5)	0.028
Ex-smoker	691(20.4)	128(13.2)		148(16.1)	40(9.9)		230(20.8)	36(15.5)		313(28.0)	52(18.0)	
Current smoker	641(22.8)	192(27.6)		212(23.9)	91(32.0)		277(25.4)	62(27.0)		152(14.9)	39(17.5)	
Non-response	45	15		7	4		16	2		22	9	
Alcohol, n(%)												
No drinker	1537(40.2)	422(41.8)	0.471	328(31.3)	123(33.8)	0.508	576(40.9)	104(37.7)	0.38	633(56.0)	195(68.0)	< 0.001
Current drinker	1838(59.8)	459(58.2)		651(68.8)	215(66.2)		714(59.1)	153(62.3)		473(44.0)	91(32.0)	
Non-response	35	7		3	0		13	1		19	6	
Physical activity, n(%)												
No	1552(41.5)	445(45.5)	0.074	366(34.5)	132(36.1)	0.646	571(41.9)	131(48.9)	0.072	615(54.3)	182(64.0)	0.015
Yes	1845(58.5)	435(54.5)		616(65.5)	206(63.9)		728(58.1)	126(51.1)		501(45.7)	103(36.0)	
Non-response	13	8		0	0		4	1		9	7	
BMI (kg/m^2^), n(%)												
Underweight	123(4.0)	71(8.2)	< 0.001	72(7.1)	42(11.7)	0.013	27(2.0)	16(5.2)	0.014	24(2.2)	13(4.3)	0.296
Normal weight	2202(64.4)	568(62.9)		642(65.8)	213(60.3)		853(64.0)	173(67.7)		707(62.5)	182(62.1)	
Overweight	951(27.7)	205(23.5)		228(22.9)	60(20.6)		371(29.9)	59(23.5)		352(31.8)	86(30.7)	
Obese	134(4.0)	44(5.3)		40(4.1)	23(7.4)		52(4.1)	10(3.6)		42(3.4)	11(2.9)	
Diagnosis of depression, n(%)												
No	3315(97.7)	780(86.8)	< 0.001	973(99.2)	308(88.4)	< 0.001	1267(97.4)	221(83.8)	< 0.001	1075(95.7)	251(87.2)	< 0.001
Yes	95(2.2)	108(13.2)		9(0.8)	30(11.6)		36(2.6)	37(16.2)		50(4.3)	41(12.8)	
Handgrip (kg), mean(SD)												
Right	33.52(0.20)	30.16(0.41)	< 0.001	35.38(0.36)	32.03(0.61)	< 0.001	34.47(0.27)	31.09(0.64)	< 0.001	27.84(0.36)	24.21(0.66)	< 0.001
Left	31.84(0.19)	28.62(0.39)	< 0.001	33.49(0.35)	30.39(0.61)	< 0.001	32.80(0.27)	29.55(0.63)	< 0.001	26.57(0.35)	22.92(0.61)	< 0.001
Relative handgrip (kg/kg/m^2^), mean(SD)												
Right	1.43(0.01)	1.31(0.02)	< 0.001	1.54(0.01)	1.40(0.03)	< 0.001	1.44(0.01)	1.34(0.03)	0.001	1.17(0.02)	1.05(0.03)	< 0.001
Left	1.35(0.01)	1.24(0.02)	< 0.001	1.45(0.01)	1.33(0.03)	< 0.001	1.37(0.01)	1.28(0.03)	0.001	1.12(0.01)	0.99(0.03)	< 0.001

*Abbreviations*: *BMI* body mass index, *PHQ-9* Patient Health Questionnaire, *SD* standard deviation, *Q* quartile

^*^ χ^2^ test and Student’s *t*-test were used for categorical (n(%)) and continuous variables (mean(SD)), respectively

**Fig. 2:**
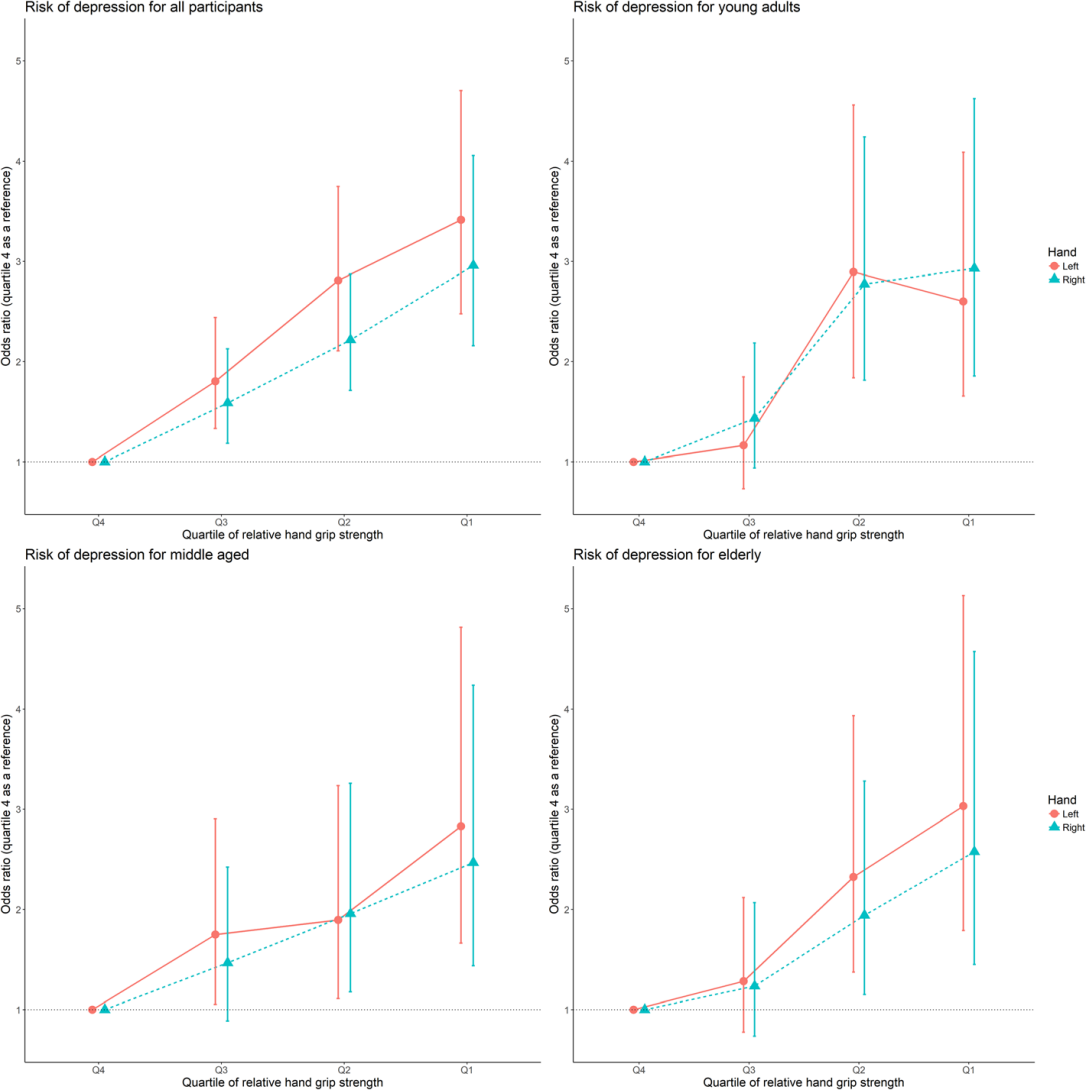
The association between quartile of relative handgrip strength and risk of depressive symptoms for age group using logistic regression Adjusted for age, sex, household income, alcohol consumption, smoking, body mass index, and physical activity. Abbreviations: Q, quartile. Quartile: All participants: Quartile 1 (right: < 0.994; left: < 0.939), Quartile 2 (right: ≥0.994, < 1.259; left: ≥0.994, < 1.191), Quartile 3 (right: ≥1.259, < 1.598; left: ≥1.191, < 1.522), Quartile 4 (right: ≥1.598; left: ≥1.522). Quartile: young adults: Quartile 1 (right: < 1.117; left: < 1.049), Quartile 2 (right: ≥1.117, < 1.368; left: ≥1.049, < 1.286), Quartile 3 (right: ≥1.368, < 1.739; left: ≥1.286, < 1.647), Quartile 4 (right: ≥1.739; left: ≥1.647). Quartile: middle aged adults: Quartile 1 (right: < 1.063; left: < 0.996), Quartile 2 (right: ≥1.063, < 1.288; left: ≥0.996, < 1.232), Quartile 3 (right: ≥1.288, < 1.649; left: ≥1.232, < 1.571), Quartile 4 (right: ≥1.649; left: ≥1.571). Quartile: elderly: Quartile 1 (right: < 0.823; left: < 0.774), Quartile 2 (right: ≥0.823, < 1.088; left: ≥0.774, < 1.023), Quartile 3 (right: ≥1.088, < 1.422; left: ≥1.023, < 1.362), Quartile 4 (right: ≥1.422; left: ≥1.362)

In both model 1(adjustment for age, sex) and model 2(adjustment for age, sex, household income, physical activity, alcohol consumption, smoking, BMI, and physical activity), relative handgrip strength was inversely associated with PHQ-9 (*P* < 0.05) (Table 2). Results observed using dichotomized outcomes for PHQ-9 (normal vs. depression) are shown in Fig. 2 in all participants and age group (young adults, middle-aged, and elderly). Among individuals who had the lowest quartile of handgrip strength, the OR of having depression was 2.96 (95% confidence interval [CI] 2.16–4.06) and 3.41 (95% CI 2.48–4.70) compared to those with the highest quartile in the right and left hands, respectively for all participants. By age group, the OR of having depression with the lowest quartile were 2.47–3.03 (*p* < 0.05) compared to the highest quartile (Fig. 2).

**Table 2:** The association between continuous handgrip strength value and depressive symptoms (PHQ-9) using multiple linear regression

	Relative right hand (kg/kg/m^2^)				Relative left hand (kg/kg/m^2^)			
	Model 1		Model 2		Model 1		Model 2	
	β (95% CI)	*P*	β (95% CI)	*P*	β (95% CI)	*P*	β (95% CI)	*P*
PHQ-9	−0.72(−1.23,-0.22)	**0.005**	−0.76(−1.26,-0.25)	**0.004**	−0.69(−1.22,-0.16)	**0.012**	− 0.83(− 1.36, − 0.29)	**0.003**

Bold numbers highlight the statistical significance

Statistical models are as follows: Model 1: adjusted for age, sex; Model 2: Model 1, plus household income, alcohol consumption, smoking, body mass index, and physical activity

*Abbreviations*: *CI* confidence interval, *PHQ-9* Patient Health Questionnaire

**Fig. 3:**
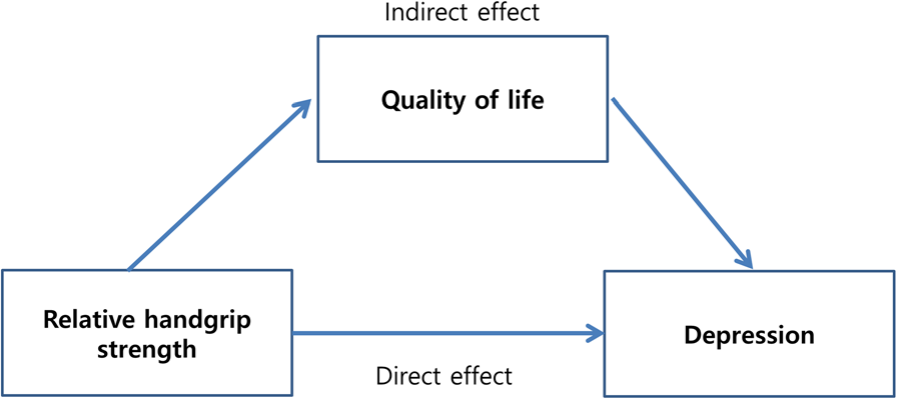
Mediation analysis

We showed Fig. 3 for mediation analysis. Regarding relative handgrip strength and depression, 56% (95% CI: 40.5–90.4) and 52% (95% CI: 38.1–80.9) of the total effect of handgrip strength on depression were explained through the mediation pathway of EQ5D, right and left handgrip strength, respectively.

In sensitivity analysis, relative handgrip strength was inversely associated with depressive symptoms by sex (except for relative right handgrip in women) (Additional file 2: Table S2).

## Discussion

The major findings of this study are that lower handgrip strength is significantly associated with having depressive symptoms in a large population-based sample, according to a self-administered questionnaire (PHQ-9). In particular, our results reveal that, compared to those with the highest quartile of relative handgrip strength, participants with the lowest quartiles were at 2.5–3.4 times the higher risk of depressive symptoms, respectively in all adults and age group (young adult, middle-aged, and elderly). Results also revealed that quality of life has a mediation effect of approximately 50% in the relationships between handgrip strength and depression.

Our prevalence of depression is consistent with previous studies (17.3–28.8%) [23]. The Korean prevalence of depression in our data was similar to that in the United States, 20.1% (PHQ-9 score ≥ 5) using National Health and Nutrition Examination Survey (NHANES) data, 2005–2008 [24].

Our results are consistent with previous studies. According to the cohort study, in Japanese adults aged 40–79 years, people with low grip strength had higher OR of depressive symptoms [8]. Lino et al. [7] found that decreased muscle strength is significantly associated with depression in Brazilians aged 60 years or older. However, another study did not find significant differences between depressed and non-depressed elderly people in terms of handgrip strength [25].

In some study, the risk of depression was higher in those who were obese and whose grip was reduced by 1SD over 4 years compare to those who were in normal weight and maintained constant handgrip [9]. In our study, however, the interaction of obesity and handgrip strength on depressive symptoms was not significant (results not shown).

A recent study showed that muscle strength was significantly positively associated with quality of life in elderly people [26]. Another study also demonstrated that handgrip strength was related to physical and mental component summary scores of quality of life [27]. Fukumori et al. [8] suggested that there is growing evidence of handgrip strength being closely associated with decreased quality of life, so handgrip strength affected mental health via quality of life. To our knowledge, this is the first study to examine the mediation effects of quality of life on the relationship between handgrip strength and depressive symptoms.

The present study has important strength, including the large size nationally representative sample; our results evaluate the effect of muscular strength on depression. This is first report performed stratification analysis by age group (young adults, middle-aged, and elderly) and found quality of life had a medication effect in relationship between relative handgrip strength and depression in adults. In addition, our results are especially important in terms of healthy aging. Similar to a previous study [28], there was a significant negative relationship between age group and handgrip strength in our data (right hand: *r* = − 0.016, *p* < 0.001, left hand: *r* = − 0.014, *p* < 0.001). Handgrip strength decreased with aging and appeared to be a predictor of development of geriatric disease, and lifelong management of handgrip indicates a great potential for promoting healthy aging [29]. In practice, including handgrip strength in medical screenings may aid in identification of potential high-risk individuals for depression.

This study had several limitations. First, because of its cross-sectional study design, it is difficult to verify the causal relationship between handgrip strength and depression; a reverse relationship was not excluded. For example, some studies have found that depression and anxiety were associated with poor handgrip strength during a six-year follow-up [30]. It is possible for psychopathology to degrade physical function [31]. Second, our data were restricted to the Korean population, so the results may not be generalizable to populations of different ethnicities. Third, we don’t have data on family history of depression that may affect depressive symptoms. Fourth, we had no information about the size of the hand that could affect handgrip strength. Fifth, questionnaire on acute social problems that could affect depression have not been investigated. Finally, due to the limitations of the questionnaire items, it was impossible to divide the subjects into physical activity recommendation according to the American College of Sports Medicine.

## Conclusions

Our study showed the association between muscular strength and depression. We found that the increased muscular strength may reduce the risk of becoming depressive symptom in all subjects and age groups (young adults, middle-aged and elderly), and our results suggest that quality of life mediates the relationship between handgrip strength and depression in adults. Future longitudinal studies focusing on proper management of lower handgrip strength in adults will help determine the clinical implications of these findings.

## Additional files

Additional file 1: **Table S1.** Weight status of the participants for each sex, for each age group and according to depression. (DOCX 18 kb)

Additional file 2: **Table S2.** The association between continuous handgrip strength value and depressive symptoms for sex using multiple linear regression. (DOCX 15 kb)

## Abbreviations

BMI: Body mass index
CI: Confidence interval
EQ5D: EuroQol five-dimension questionnaire
KNHANES: Korea National Health and Nutrition Examination Survey
NHANES: National Health and Nutrition Examination Survey
OECD: Organization for economic cooperation and development
OR: Odds ratios
PHQ-9: Patient health questionnaire 9
SD: Standard deviation
