# Supplementary material for: The association between muscular strength and depression in Korean adults: a cross-sectional analysis of the sixth Korea National Health and Nutrition Examination Survey (KNHANES VI) 2014
Source: BMC Public Health. 2018 Sep 15;18:1123. doi: 10.1186/s12889-018-6030-4 (PMC6139143; doi:10.1186/s12889-018-6030-4)
Supplement: Supplementary file 1 — Table S1. Weight status of the participants for each sex, for each age group and according to depression. (DOCX 18 kb) [file 12889_2018_6030_MOESM1_ESM.docx]

Table S1. Weight status of the participants for each sex, for each age group and according to depression

|  | Young adult | |  |  | Middle aged | |  |  | elderly |  |  |  |
| --- | --- | --- | --- | --- | --- | --- | --- | --- | --- | --- | --- | --- |
|  | Men(552) |  | Women(n=768) | | Men(n=658) | | Women(n=903) | | Men(n=650) | | Women(n=767) | |
|  | PHQ-9<5 | PHQ-9≥5 | PHQ-9<5 | PHQ-9≥5 | PHQ-9<5 | PHQ-9≥5 | PHQ-9<5 | PHQ-9≥5 | PHQ-9<5 | PHQ-9≥5 | PHQ-9<5 | PHQ-9≥5 |
| Underweight | 12(2.7) | 7(6.5) | 60(11.2) | 35(15.2) | 6(1.0) | 3(3.8) | 21(2.9) | 13(7.3) | 20(3.6) | 7(7.8) | 4(0.7) | 6(3.0) |
| Normal weight | 250(56.3) | 61(56.5) | 392(72.9) | 152(66.1) | 336(58.1) | 48(60.0) | 517(71.3) | 125(70.2) | 366(65.4) | 60(66.7) | 341(60.4) | 122(60.4) |
| Overweight | 151(34.0) | 32(29.6) | 77(14.3) | 28(12.2) | 211(36.5) | 25(31.3) | 160(22.1) | 34(19.1) | 162(28.9) | 19(21.1) | 190(33.6) | 67(33.2) |
| Obese | 31(7.0) | 8(7.4) | 9(1.7) | 15(6.5) | 25(4.3) | 4(5.0) | 27(3.7) | 6(3.4) | 12(2.1) | 4(4.4) | 30(5.3) | 7(3.5) |

Participants into underweight (BMI <18.5), normal weight (18.5≤BMI<25), overweight (25≤BMI<30), obese (30≤BMI)

Abbreviations: BMI, body mass index; PHQ-9, Patient Health Questionnaire
