# Supplementary material for: The association between muscular strength and depression in Korean adults: a cross-sectional analysis of the sixth Korea National Health and Nutrition Examination Survey (KNHANES VI) 2014
Source: BMC Public Health. 2018 Sep 15;18:1123. doi: 10.1186/s12889-018-6030-4 (PMC6139143; doi:10.1186/s12889-018-6030-4)
Supplement: Supplementary file 2 — Table S2. The association between continuous handgrip strength value and depressive symptoms for sex using multiple linear regression. (DOCX 15 kb) [file 12889_2018_6030_MOESM2_ESM.docx]

Table S2. The association between continuous handgrip strength value and depressive symptoms for sex using multiple linear regression

|  | Men |  |  |  | Women |  |  |  |
| --- | --- | --- | --- | --- | --- | --- | --- | --- |
|  | Model 1 |  | Model 2 |  | Model 1 |  | Model 2 |  |
|  | β (95% CI) | *P* | β (95% CI) | *P* | β (95% CI) | *P* | β (95% CI) | *P* |
| Relative right hand | |  |  |  |  |  |  |  |
|  | -0.56(-1.06,-0.06) | **0.029** | -0.65(-1.18,-0.13) | **0.016** | -1.07(-2.07,-0.08) | **0.035** | -0.88(-1.94,0.17) | 0.101 |
| Relative left hand | |  |  |  |  |  |  |  |
|  | -0.46(-0.99,0.06) | 0.083 | -0.61(-1.18,-0.03) | **0.038** | -1.16(-2.16,-0.15) | **0.024** | -1.14(-2.23,-0.05) | **0.040** |

Abbreviations: CI, confidence interval; PHQ-9, Patient Health Questionnaire.

Bold numbers highlight the statistical significance.

Statistical models are as follows: Model 1: adjusted for age; Model 2: Model 1, plus household income, alcohol consumption, lifetime smoking, body mass index, and physical activity
